# The reinforcing effect of anti-caries treatments on acidic challenge resistance of irradiated enamel

**DOI:** 10.1371/journal.pone.0350046

**Published:** 2026-05-29

**Authors:** Fereshteh Shafiei, Iman Khaleghi, Mansour Ansari, Esmaeil Mirzaei, Maryam S. Tavangar

**Affiliations:** 1 Oral and Dental Disease Research Center, Department of Operative Dentistry, School of Dentistry, Shiraz University of Medical Sciences, Shiraz, Iran; 2 Department of Operative Dentistry, Lorestan University of Medical Sciences, Khorramabad, Iran; 3 Radiation Oncology Department, Medical School, Shiraz University of Medical Sciences, Shiraz, Iran; 4 Department of Medical Nanotechnology, School of Advanced Medical Sciences and Technologies, Shiraz University of Medical Sciences, Shiraz, Iran‌‌; International Medical University, MALAYSIA

## Abstract

**Aim:**

To investigate the effect of applying the following anticaries treatments on the surface microhardness (SMH) of irradiated enamel after pH-cycling: Nanohydroxyapatite (nano-HA), resin infiltration (RI), combination of nano-HA and RI, or curcumin-loaded mesoporous nano-silica (Cur/MSN).

**Methodology:**

In this in vitro experimental study, 30 sound molars were mesiodistally sectioned into two halves, yielding 60 enamel samples, and randomly divided into six groups (n = 10). Group 1 was kept as non-irradiated. The samples from the other five groups were subjected to fractionated X-ray radiation for 6 weeks, with a total dose of 60 Gy, delivered in 2 Gy fractions 5 days per week. After irradiation, each group was processed as follows: 2) no treatment; 3) treated with 15% nano-HA; 4) treated with RI; 5) treated with a combination of 15% nano-HA and RI; 6) treated with Cur/MSN (3 mg/ml). The initial SMH (SMH_0_) was measured after irradiation and the final (SMH_1_) after anticaries treatments and pH cycling. pH cycling consisted of a 3-hour immersion in a demineralizing solution, followed by 21 hours in a remineralization solution at 37 °C for 5 days. The surface micromorphology of irradiated groups after pH-cycling was assessed.

**Results:**

Mean [±SD] SMH_0_ of irradiated enamel was significantly lower than that of the non-irradiated one (330.7 ± 23.6 vs 262.9 ± 28.8; P < 0.001). Irradiated enamels treated with all anti-caries treatments showed a significantly higher SMH_1_ compared to those of no-treatment (192.2 ± 28.7; all P < 0.01). SMH_1_ of Ir/nano-HA (302.2 ± 16.4) or Ir/nano-HA + RI (305.4 ± 22.1) groups were higher than those of Ir/RI (245.5 ± 35.1; P < 0.001) and not different from Ir/Cur/MSN group (285.9 ± 16.4; P = 0.638 and 0.435 respectively). The increase in SMH_1_ compared to SMH_0_ (ΔSMH) was significant only in Ir/nano-HA (39.0; P = 0.003) and Ir/nano-HA + RI groups (42.9; P = 0.001).

**Conclusion:**

Treatment with nano-HA and a combination of nano-HA with RI demonstrated significant potential to improve the resistance of the irradiated enamel surface to acidic challenges.

## Introduction

Head and neck cancer has been reported as the seventh most common cancer in the world [1]. According to the World Health Organization (WHO), most patients involved in head and neck cancer receive radiotherapy (RT) as a treatment option [2]. Depending on tumor development, RT protocol is between 50 and 70 grays (Gy) in a fractional daily dose of 2 Gy [3]. This therapeutic radiation can cause complications, such as destructive effects on tissues adjacent to the tumor. The primary and prevalent complication in the head and neck region is radiation caries, resulting from the direct and indirect effects of RT [4,5]. The primary etiological factors are damage to the salivary glands, the resulting reduced flow rate of saliva, and its protective effects [6]. In addition, suboptimal oral hygiene, changes in oral bacterial flora, and an increase in a soft or carbohydrate-rich food regimen are contributing factors [7–9].

Some investigators have shown that RT reduces microhardness and mechanical properties, leading to micromorphological and chemical changes in enamel [8,10–13]. On the contrary, an earlier in-situ study revealed that RT did not obviously affect the microhardness of enamel, suggesting a more chemical rather than physical alteration in its microstructure following RT [14]. Hence, this issue has remained controversial, and a similar debate can be noticed regarding caries susceptibility of irradiated enamel. While an in situ study indicated no difference in caries susceptibility and initial demineralization between RT and non-RT enamel [15], another study showed increased susceptibility to enamel demineralization following xerostomia, accompanied by a decrease in pH in the oral environment [12]. In a previous study, it has been proposed that RT-induced enamel is more significantly affected by demineralization than remineralization [16]. On the contrary, some have reported that in vitro demineralization or in situ remineralization of enamel was not affected by RT [17].

Given the high survival and cure rates in patients with head and neck cancers, preserving and reinforcing enamel damaged by radiotherapy is essential to protect against acid attack. Effective reinforcing or protective measures are recommended for damaged enamel before, during, and after the RT procedures [18]. Considering the rapid onset of radiation-induced caries, implementing caries-preventive strategies after RT would be highly beneficial [5,11]. This could help prevent dental breakdown after RT. On this basis, several studies demonstrated that anti-caries/remineralizing agents could promote tooth structure remineralization following RT [10,16,19]. The Use of 1% casein phosphopeptide-amorphous calcium phosphate (CPP-ACP), bioactive glass, chitosan, fluoride compounds, 30% silver diamine fluoride (SDF), and 4% Titanium tetrafluoride (TiF_4_) has been reported to partially ameliorate the adverse effects of RT on enamel [10,16,20–22].

Resin infiltration (RI) is a promising minimally invasive technique primarily applied to non-cavitated, initial enamel caries to arrest the progression of demineralization [23,24]. This technique uses a low-viscosity resin with a high penetration coefficient that infiltrates enamel porosities caused by mineral loss, thereby mechanically stabilizing the lesion [25–27]. RI treatment could further reduce demineralization [27], but it could not remineralize the infiltrated enamel. In an investigation, researchers used varnish fluoride and CCP-ACP on irradiated enamel, and RI alone or in combination with fluoride and CCP-ACP. RI and the latter combination were revealed to be the most effective in initially restoring the destruction of enamel caused by RT [20]. Their study did not assess the effect of these substances on the acid resistance of irradiated enamel. Consequently, the impact of RI alone or combined with a remineralizing agent on the acid resistance of irradiated enamel may be a matter of investigation.

Nanohydroxyapatite (nano-HA) is a biomimetic material with biocompatibility and bioactivity properties [26]. It acts as a reservoir of calcium and phosphate that could reduce demineralization and enhance remineralization of incipient demineralized enamel [28,29]. This enhanced bioactivity is attributed to a crystal structure and morphology similar to that of dental hard tissue apatite [29,30]. Nano-HA application to irradiated enamel may enhance its resistance to an acidic challenge.

Curcumin (Cur) is a natural hydrophobic polyphenol compound with unique properties. Because of its anticancer, antioxidant, anti-inflammatory, antibacterial, and collagen-crosslinking effects, it is widely studied for the prevention and treatment of various illnesses [31,32]. The antioxidant and cross-linking properties of Cur may be beneficial for the tooth structure after RT [33]. Mesomorphous silica nanoparticles (MSN) with large pore volumes, high internal surface areas, and low cost have been widely used in the biomedical field. These particles are ideal nano-carriers for drug delivery with sustained release [34,35]. Loading Cur onto MSN will likely facilitate its delivery to substructures within irradiated enamel.

Since RT may increase the sensitivity of enamel to demineralization [35], the use of remineralizing/anticaries agents was assumed to be effective on irradiated enamel. There was little information on the protective effects of RI, nano-HA, their combination, and Curcumin-loaded MSN on the acid-challenge resistance of irradiated enamel. Accordingly, the current study aims to assess the null hypothesis that different preventive methods—RI, nano-HA, RI + nano-HA, and Cur/MSN— would not influence the microhardness and surface micromorphology of irradiated enamel evaluated after pH cycling.

## Materials and methods

**Fig 1:**
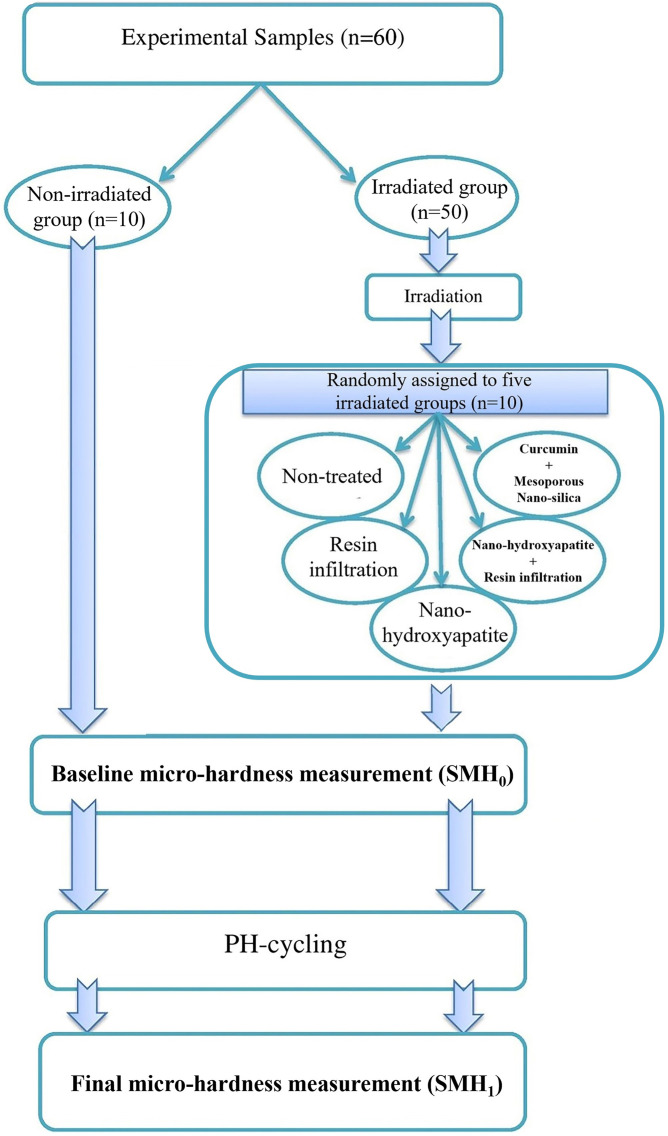
Flowchart diagram of the study.

In this in vitro experimental study conducted on extracted human teeth, enamel samples were randomly allocated to six study groups consisting of one non-irradiated group (n = 10), which served as control, vs five irradiated groups (n = 5 groups × 10 specimens = 50 samples), including four groups treated with different anti-caries agents in addition to one untreated group. So, there are two study factors used for group labeling: irradiation and anticaries treatment agent. This resulted in six experimental conditions. The primary response variables were the initial surface microhardness (SMH_0_), the surface microhardness evaluated after surface-treatment and pH-cycling (SMH_1_), and the calculated change in surface microhardness (ΔSMH = SMH_1_ − SMH_0_). Secondary outcomes included qualitative assessment of enamel surface micromorphology using scanning electron microscopy (SEM). The study flowchart is presented in Fig 1.

This study was approved by the local Research Ethics Committee (ethical approval number IR.SUMS.DENTAL.REC.1402.075- approved at 2023-07-05.). Thirty sound third molars extracted due to orthodontic reasons were collected from August 2023 to November 2023 (The Human Participants Research Checklist is provided as supplementary material S1 File). All the patients gave informed written consent.

After removing their roots at the cemento-enamel junction (CEJ) using a water-cooled diamond saw disc (Buehler; LakeBluff, IL, USA), the teeth were cleaned and disinfected in chloramine T solution (Sigma-Aldrich; Sigma-Aldrich, St. Louis, MO, USA) for 4 weeks. Following assessment with a magnifying glass, none of them has a crack or any signs of enamel hypoplasia or fluorosis.

### Sample preparation

The 30 selected crowns were sectioned longitudinally in the mesiodistal direction using a diamond saw under water cooling into two halves. The enamel of both buccal and lingual aspects of each tooth was cut to obtain 60 enamel slabs with a thickness of 2 mm and a width of 4 mm. After that, they were mounted with a self-cured acrylic resin (Acropars; Marlik, Tehran, Tehran, Iran) horizontally. The enamel surfaces were then polished using silicon carbide papers (KEEJEA; Seoul, Seoul, Republic of Korea) with an increasing grit sequence from 800 to 7000 grit, under running water, to provide a flat enamel surface.

#### Irradiation protocol.

The teeth were subjected to fractionated radiation. Fifty teeth assigned to receive radiation were placed in plastic vials (Pole ideal Pars co; Tehran, Tehran, Iran) containing distilled water in a way to receive a uniform radiation dose. A total of 60 Gy irradiation, consisting of 2 Gy exposure applied 5 days per week (for 6 weeks) with 6 MV X-rays from a linear accelerator (Vital Beam; Varian, Palo Alto, CA, USA), was applied to the samples [21]. During radiotherapy, the samples were stored in distilled water, which was changed daily, because submersion in artificial saliva could impede effective irradiation due to its viscosity and high ion concentration [36]. The beam geometry and radiation dose were selected similarly to those used in the beam setup and prescription dose for patients with head and neck tumors. Plans were performed using TPS software (Eclipse version 15; Varian, Palo Alto, CA, USA). Additionally, the samples from the non-irradiated groups were stored in distilled water, which was renewed daily, to simulate conditions similar to those of the irradiated groups.

#### Experimental grouping.

Fig 1 illustrates the study’s flow diagram. The prepared samples were randomly divided into six groups (n = 10) as follows:

1) Non-Irradiated (non-Ir); the enamel was not irradiated or treated by any agents.
2) Irradiated (Ir); the enamel was irradiated but not treated with any agents.
3) Irradiated/resin-infiltration (Ir/RI); the enamel was irradiated and then etched with 15% HCl (Icon-Etch; DMG, Hamburg, Hamburg, Germany) for 2 min, rinsed for 30 seconds, and dehydrated with 10% ethanol for 30 seconds. Resin infiltration (Icon resin, DMG, Hamburg, Hamburg, Germany) was applied using a microbrush for 3 minutes. Excess resin was gently removed, and the resin layer was light-cured using an LED light-curing unit at an intensity of 1200 mW/cm^2^ (BlueLEX GT-1200; Monitex Industrial, New Taipei, Taiwan) for 40 seconds. The additional resin layer was again used for 1 minute and light-cured for 40 seconds. The manufacturer’s instructions were followed in this procedure.
4) Irradiated/nano-HA (Ir/nano-HA); the enamel was irradiated and then treated with a 15% solution of nano-HA for ten minutes. This solution was prepared by dissolving nano-HA (Merck; Merck, Darmstadt, Hessen, Germany) in a 1:1 mixture of distilled water and acetone [29]. The nanoparticle size analyzer (Horiba; Horiba, Kyoto, Kyoto, Japan) was used to measure particle size. The mean size was recorded as 12.55 nm.
5) Irradiation/nano-HA + resin infiltration (Ir/nano-HA + RI); the enamel was irradiated and then etched with 15% HCl for 2 minutes, rinsed for 30 seconds, and air-dried. Then, a nano-HA solution was applied. After that, Icon resin was applied and light-cured as described above.
6) Irradiated/curcumin/MSN (Ir/Cur/MSN); the enamel was irradiated and treated with curcumin loaded in mesoporous silica nanoparticles (3 mg/mL suspension), which was activated with an LED light-cure unit with light intensity at 1200 mW/cm^2^.

MSN was prepared according to the reference procedure with slight modifications [37]. Briefly, 0.5 g of Cetyltrimethylammonium bromide (CTAB) (Sigma-Aldrich; Sigma-Aldrich, St. Louis, MO, USA) was dissolved in 250 mL of distilled water under ultrasonic agitation to ensure complete solubilization. Subsequently, 2 mL of 2 M NaOH (Sigma-Aldrich; Sigma-Aldrich, St. Louis, MO, USA) was added to the mixture, and the mixture was heated to 80 °C under continuous stirring. Once the temperature stabilized, 5.0 mL of Tetraethyl orthosilicate (TEOS) (Sigma-Aldrich; Sigma-Aldrich, St. Louis, MO, USA) was added dropwise to the reaction mixture and maintained at 80 °C for 2 hours to enable the formation of MSNs. The resulting precipitate was collected by centrifugation at 9000 rpm for 15 minutes, followed by sequential washing with deionized water and ethanol. The final product was dried under vacuum for further use.

To encapsulate curcumin in MSNs, 100 mg of curcumin (Sigma-Aldrich; St. Louis, MO, USA) was dissolved in 10 mL of absolute ethanol (Dr Mojallali; Tehran, Tehran, Iran). Then, 1.5 g of previously synthesized MSNs was added to the curcumin solution. The mixture was stirred at room temperature in the dark for 24 hours to facilitate loading. After incubation, the mixture was centrifuged at 10,000 rpm for 30 minutes to remove any unencapsulated curcumin. The supernatant was retained for subsequent quantification of curcumin loading. The resulting pellet was washed repeatedly with distilled water and ethanol and then dried under vacuum. Finally, a 3 mg/mL suspension of MSN-Cur was prepared.

#### Characterization.

The morphological characteristics of the synthesized nanoparticles were examined using transmission electron microscopy (TEM) (Carl Zeiss EM10C 100 kV; Carl Zeiss, Oberkochen, Germany) operated at an acceleration voltage of 100 kV. Structural analysis of the MSNs was performed using low-angle X-ray diffraction (XRD) on a diffractometer (AW-XDM 300; ASENWARE, Shenzhen, Guangdong, China) equipped with Cu Kα radiation. Diffraction data were collected in the range of 0.5–10° 2θ at a resolution of 0.1° 2θ. Functional group analysis and confirmation of curcumin loading were performed using Fourier Transform Infrared (FTIR) spectroscopy in the range of 400–4000 cm ⁻ ¹ (Bruker Vertex 70 and Tensor II spectrometer; Bruker Optics, Ettlingen, Baden-Württemberg, Germany).

#### Curcumin loading content.

To determine the curcumin loading content, the absorbance of different concentrations of curcumin in ethanol was first measured at 425 nm using a UV–Vis spectrophotometer (Genesys; Thermo Fisher Scientific; Shanghai, Shanghai Municipality, China) to establish a calibration curve. After loading, the MSN-Cur suspension was centrifuged at 10,000 rpm for 30 minutes at 4 °C in a refrigerated centrifuge. The supernatant, containing free (unencapsulated) curcumin, was carefully collected, and its absorbance was measured at 425 nm.

The amount of free curcumin was calculated using the standard calibration curve. The loading content (LC%) of curcumin into MSNs was determined using the following equation:

Loading Content (%) = (Total amount of Cur – the amount of free Cur in supernatant/Total amount of Cur-MSN) ×100%

#### Initial surface microhardness (SMH_0_).

Initially, the SMH of the non-irradiated and five irradiated groups before applying different treatments was determined using a microhardness tester (MHV-1000; SCTMC; Shanghai, Shanghai Municipality, China) with a Vickers diamond indenter under a load of 25 g for 10 seconds at each test point [38]. The measurement was performed at three different points, and the mean value was recorded as the Vickers hardness number in kg/mm². These values in this step were considered as SMH_0_.

#### pH-cycling and final microhardness (SMH_1_) assessment.

All six groups were subjected to a pH-cycling regimen following treatments on four irradiated groups. This consisted of a 3-hour immersion in a demineralizing solution, followed by a 21-hour immersion in a remineralization solution at 37 °C for five days [19,39].

The samples were rinsed in deionized water between two solution exchanges. The composition of the demineralizing solution was 0.75 mM acetate buffer, containing 2.2 mM calcium (CaCl_2_), 2.2 mM phosphate (NaH_2_PO_4_), and 0.03 F μg/ml. The solution had a pH of 4.3, and it was applied at a rate of 6.36 ml/mm^2^ of exposed enamel area. The chemical composition of the remineralizing solution was 1.5 mM calcium, 0.9 mM phosphate, 0.15 M KCl, 0.05 F μg/mL, and 20 mM cacodylate buffer at a pH of 7.0, applied in the proportion of 3.18 mL/mm². Both solutions contained thymol crystals to avoid microbial growth [39]. All the materials were obtained from a same company (Sigma-Aldrich; Sigma-Aldrich, St. Louis, MO, USA).

Following this acidic challenge, the microhardness of six groups was measured as described in the previous part. The recorded value was considered SMH_1_. All procedures in this study were performed by the same trained operator who was blinded to the specimen groupings.

Then, the mean difference (ΔSMH) was determined:


$$\begin{array}{c} \Delta \text{SMH}=\text{SM}{\text{H}}_{\text{1}}-\text{SM}{\text{H}}_{\text{0}} \end{array}$$

#### Surface micromorphology analysis by scanning electron microscope (SEM).

One specimen from each irradiated group, after pH cycling, was selected for examination using a scanning electron microscope (VEGA III; TESCAN; Brno, South Moravia, Czech Republic) at magnifications of ×2000 and ×5000. The samples were affixed to metal mounts, dehydrated by vacuum, and sputter coating was performed with a gold coating using a sputter coater (SCD 050; Balzers; Schaan, Liechtenstein).

#### Statistical analysis.

Following confirmation of data normality using the Kolmogorov-Smirnov test, one-way ANOVA was used to compare SMH_0_ and SMH_1_ within each study group. A pair-wise comparison was then conducted using the post hoc Tukey test. An independent-samples t-test was used to compare values between SMH_0_ and SMH_1_ within each study group. All analyses were performed using a statistical analysis software (SPSS version 17; SPSS; Chicago, IL, USA) (α = 0.05). The raw data for the study are provided in supplementary material S2 File.

## Results

### TEM and XRD analysis

**Fig 2:**
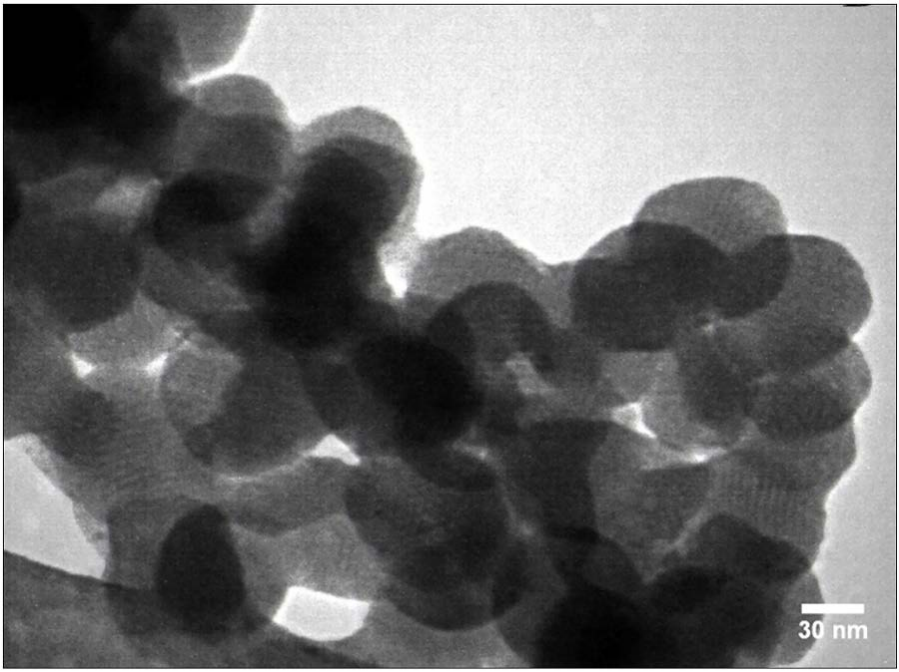
Transmission electron microscopy (TEM) images of mesoporous silica nanoparticles (magnification = 100,000 X).

TEM images of MSN are shown in Fig 2. TEM analysis shows that monodispersed MSN have a spherical shape and a diameter of 70 ± 2 nm. Moreover, the image shows ordered pore-like channels on the surface of the nanoparticles, which is consistent with the XRD analysis that follows.

**Fig 3:**
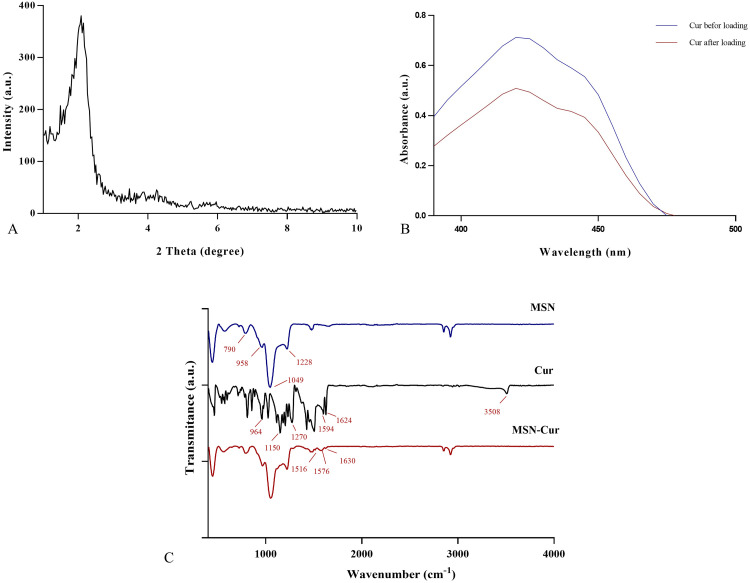
Analytical Studies on mesoporous silica nanoparticles (MSN). **A)** X-ray diffraction (XRD) analysis pattern of MSN. **B)** UV-Vis spectra of Curcumin before and after loading to MSN. **C)** Fourier Transformed Infrared (FTIR) spectroscopy (FTIR) of MSN, Curcumin, and Curcumin-loaded MSN. The FTIR spectrum of Curcumin, MSN, and Curcumin-loaded MSN in the 400−4000 cm^−1^ spectral range is shown. MSN revealed the characteristic bands observed at 1228 cm^−1^ and 1049 cm^−1^, assigned to the asymmetric stretching vibrations of Si-O-Si. The distinctive band at 790 cm^−1^ is associated with symmetric bending vibrations of Si-O-Si and the asymmetric vibration of Si-OH, indicated at 958 cm^−1^. The FTIR spectrum of free Curcumin has identified wave numbers at O-H stretching at 3508 cm^−1^, C = O vibration at 1594 and 1498 cm^−1^, C–O stretching at 1624 cm^-1^, aromatic C-O stretching at 1270 cm^−1^, aliphatic C-O stretching at 1150 cm^−1^, and C-H bending at 964 cm^−1^.

The structural properties of MSN have been determined by low-angle powder X-ray diffraction, as shown in Fig 3-A. The XRD spectrum of MSN showed a visible peak at 2θ = 2.09°; these peaks indicate a regular periodic variation in electron density, suggesting that the sample may exhibit a certain degree of structural ordering of the pores in MSN [40,41].

The absorbance of Cur before and after Cur loading to MSN is shown in Fig 3-B. The drug loading content (DLC) of Curcumin in MSN was calculated to be 9%.

### FTIR spectroscopy analysis

The synthesized MSN, Cur-loaded MSN, and Cur were characterized by FTIR analysis to identify functional groups (Fig 3-C). FTIR spectrum of Curcumin loaded MSN indicates the presence of silica and Cur; the spectrum shows that the O-H stretching peak at 3508 cm^−1^ of Cur disappeared, which can be attributed to the molecular interaction between Curcumin and MSN, as well as the trapping of Curcumin in MSN. The presence of stretching vibrations at 1516 cm^−1^, 1576 cm^−1,^ and 1630 cm^−1^ in the Curcumin-loaded MSN spectrum corresponded to the interaction between Curcumin and MSN [42–45].

#### SEM analysis.

**Fig 4:**
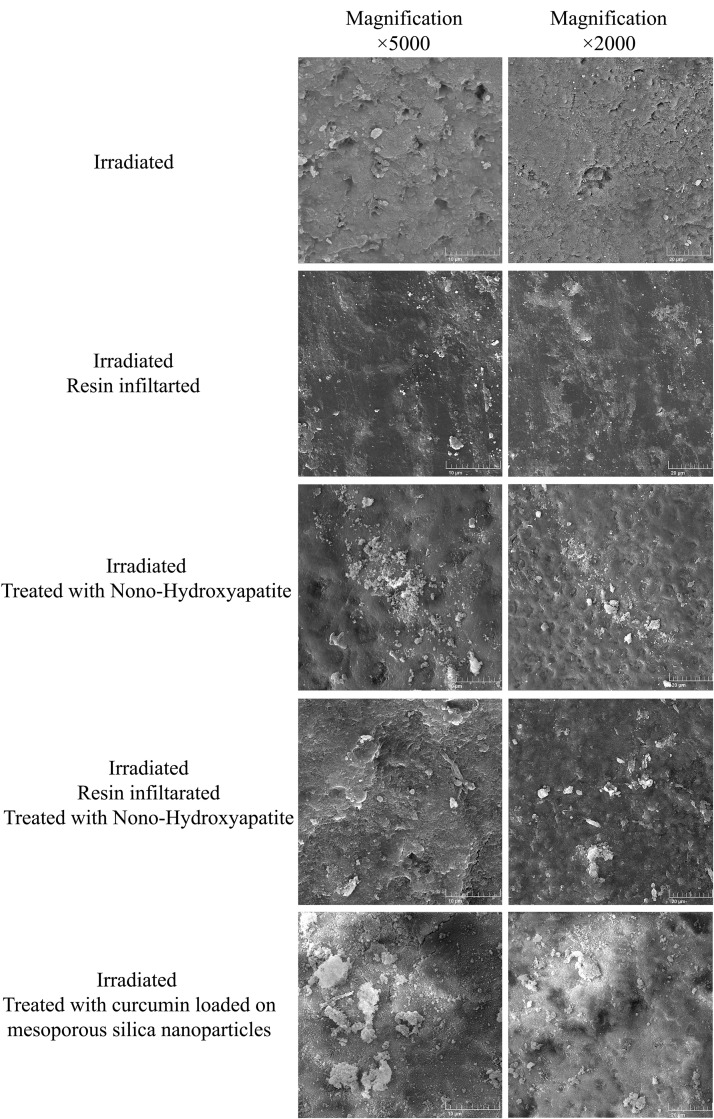
Scanning electron microscope at two magnifications of ×2000 (right column) and ×5000 (Left Column) from 5 experimental groups of the study.

SEM observation of the irradiated group indicated an uneven surface with visible micropores and microcracks. These defects were reduced across all treated groups, resulting in a more homogeneous appearance. In the Ir/Cur/MSN group, the remaining mesoporous nanoparticles were observed on the surface (Fig 4).

#### SMH analysis.

The initial and final SMH mean ± standard deviation (SD) values after treatment and pH-cycling, as well as the SMH_1_-SMH_0_ value, are presented in Table 1. The results of one-way ANOVA for each initial and final SMH showed significant differences among the six groups (P < 0.001). The SMH_0_ of the five irradiated groups was significantly lower than that of the non-Ir group (330.7 ± 23.6 vs 262.9 ± 28.8; P < 0.001). After pH cycling, all treatments showed a higher SMH_1_ compared to Non-Ir (all P < 0.001); however, the SMH_1_ of Ir/RI (245.5 ± 35.1) was significantly lower than that of Ir/nano-HA (302.2 ± 16.4; P < 0.001), Ir/nano-HA + RI (305.4 ± 22.1; P < 0.001), and Ir/Cur/MSN (285.9 ± 16.4; P = 0.004). SMH_1_ among the latter three groups did not differ statistically (all P > 0.4). Among these, the Ir/nano-HA and Ir/nano-HA + RI groups with the highest SMH_1_ were comparable to the non-Ir group (326.8 ± 15.9; P = 0.200 and 0.341, respectively). In contrast, Ir/Cur/MSN had a significantly lower value than the non-Ir group (P = 0.004), indicating a strong recovery effect of Ir/nano-HA and Ir/nano-HA + RI treatment of irradiated enamel.

**Table 1:** Initial (SMH_0_), final (SMH_1_), and change in surface microhardness (ΔSMH) values measured by Vickers’ test.

Subgroups	SMH_0_	SMH_1_	ΔSMH (SMH_1_-SMH_0_)	P-value
**Non-Ir (Group 1)**	330.70 ± 23.60^a^	326.80 ± 15.87 ^a^	−3.90^a^	0.656
**Ir (Group 2)**	262.40 ± 30.93^b^	192.20 ± 28.75 ^b^	−70.20^b^	<0.001
**Ir/RI (Group 3)**	262.50 ± 30.45^b^	245.50 ± 35.09 ^c^	−17.00^a^	0.096
**Ir/nano-HA (Group 4)**	263.20 ± 30.03^b^	302.20 ± 16.43 ^ad^	39.00^c^	0.003
**Ir/nano-HA + RI (Group 5)**	262.50 ± 29.91^b^	305.40 ± 22.13 ^ad^	42.90^c^	0.001
**Ir/Cur/MSN (Group 6)**	263.90 ± 30.45^b^	285.90 ± 16.44 ^d^	22.00^ac^	0.062
**P-value**	<0.001	<0.001	<0.001	

*Abbreviation: Cur = Curcumin, Ir = Irradiated, MSN = Mesoporous nano-silica, Nano-HA = nano-hydroxy apatite, SMH = Surface microhardness, RI = Resin Infiltration.

** Ten specimens were enrolled in each group.

*** In each column, different small letters indicate significant statistical differences. In each row, a paired T-test has been done to compare the before and after SMH value. P < 0.05 is considered statistically significant.

**** The five irradiated groups had significantly lower SMH_0_ than the non-irradiated group.

Among irradiated groups, Ir/nano-HA and Ir/nano-HA + RI showed significantly higher SMH_1_ compared to the Ir/RI and did not differ from the Ir/Cur/MSN group. The Ir group exhibited the significantly lowest SMH_1_ results. Comparing the initial and final SMH values within each group showed that final SMH was reduced numerically in Non-Ir, Ir, and Ir/RI groups, while it was increased in Ir/nano-HA, Ir/nano-HA + RI, and Ir/Cur/MSN groups. ΔSMH values for Ir/nano-HA and Ir/nano-HA + RI were significantly higher than those for the Ir/RI, Ir, and Non-Ir groups, but did not differ from the Ir/Cur/MSN group. Using the t-test showed significant differences between SMH_0_ and SMH_1_ values in the Ir, Ir/nano-HA, and Ir/nano-HA + RI groups, and no significant difference in the non-Ir, Ir/RI group, and the Ir/Cur/MSN groups.

Comparing the initial and final SMH values within each group showed that final SMH was reduced numerically in Non-Ir, Ir, and Ir/RI groups (ΔSMH<0), while it was increased in Ir/nano-HA, Ir/ nano-HA + RI, and Ir/Cur/MSN groups (ΔSMH>0). One-way ANOVA also indicated a significant difference between the ΔSMH values of the study groups (P < 0.001). The ΔSMH value for Ir/nano-HA (ΔSMH = 39) and Ir/nano-HA + RI (ΔSMH = 42.9) was significantly higher than that for the Ir/RI(ΔSMH = −17), Ir (ΔSMH = −70.2), and Non-Ir groups (ΔSMH = −3.9) (all P < 0.001), but did not differ from the Ir/Cur/MSN group (ΔSMH = 22; P = 0.814 and 0.648, respectively).

Using the t-test showed significant differences between SMH_0_ and SMH_1_ values in the Ir (P < 0.001), Ir/nano-HA (P = 0.003), and Ir/nano-HA + RI (P = 0.001) groups and no significant difference in the non-Ir (P = 0.656), Ir/RI group (P = 0.096), and the Ir/Cur/MSN (P = 0.062) groups.

## Discussion

Despite the high efficacy of RT in the treatment of head and neck cancer patients, its adverse effects on the hard tissue tooth structure are inevitable [5]. RT does range from 50 to 70 Gy, depending on tumor stage. In severe cases, a maximum RT dose of 70 Gy or more is recommended [46]. In this study, 60 Gy was applied, which is an average dose and has been used in several studies [21,38,47,48]. With increasing radiation dose, adverse effects become more severe in a way that at 60 Gy or more, a tenfold increased risk of tooth damage was reported [6]. This damage, including enamel fractures and erosions, exposes dentin, leading to rapid progression of radiation caries within 4–6 months after RT [9]. Hence, effective reinforcing or protective measures are recommended for damaged enamel before, during, and after the RT procedures [18]. In clinical situations, it might be more practical to use these measures once patients have completed all RT sessions and have been referred to dental care, as was the case in the current study for caries preventive treatment. Considering the rapid onset of radiation-induced caries, implementing caries-preventive strategies after RT was investigated by some researchers [5,11]. On the other hand, several studies suggested protective and preventive approaches before or during RT [10,16,18,20,21]. Consequently, an early and active involvement in developing preventive and therapeutic strategies, is crucial for addressing quality-of-life issues before, during, and after radiotherapy as early as possible [49].

Our study revealed that RT remarkably decreased the SMH of enamel. The altered surface micromorphology, including microcracks and pores, was also observed in our irradiated enamel samples. This reduced SMH finding is consistent with results from previously published studies that used fractional doses in the 50–70 Gy range [10–13,16,20,21,38]. A similar result was recently demonstrated with a single 60 Gy dose [22]. This reduction in enamel SMH due to RT was attributed to the formation of micro-pores and fractures in hydroxyapatite crystals, as well as to increased permeability, reduced crystallinity, and a reduced mineral content in irradiated enamel [13,48]. These chemical alterations consist of changes in Ca/P Ratios, oxygen, Magnesium, and potassium levels [21]. Besides, enamel prisms may be affected as the RT decreases their hardness and elasticity [50].

The resistance of irradiated enamel to an acid challenge in the current study was mimicked by pH cycling. This model comprises a demineralization-remineralization cycle that simulates the dynamic movement of minerals and changes in pH. Hence, it is a commonly used method to assess the anticarcinogenic potential of dental materials [10,22,51]. In previous studies, a linear correlation has been found between the microhardness value and the mineral content of enamel [9,20]. Accordingly, the SMH test has been widely used to assess the degree of enamel demineralization [52]. This test, in conjunction with SEM analysis, is essential for evaluating the demineralization and remineralization of enamel and dentin in caries research studies [20]. Therefore, SMH measurement and SEM analysis, were employed in this study along with pH cycling to investigate the surface microhardness of irradiated enamel with and without protective anti-caries agents.

In the present investigation, irradiated enamel after pH cycling showed a significant reduction in SMH compared with any other group studied. Similarly, a recent study demonstrated that radiation can decrease enamel acid resistance and increase enamel demineralization [53]. It was also reported that the deleterious effects of RT on dental hard structures appear similar to the demineralization process [54]. Several studies have reported a decrease in apatite crystallinity and an increase in the solubility of irradiated enamel in saliva at acidic pH [9,16,38,47]. Furthermore, a highly porous, more permeable enamel structure was documented in the SEM analysis of irradiated enamel [48]. It has also been reported that radiation could reduce calcium ion levels and lower the Ca/P ratio [13]. The reduced mineral content and increased permeability may explain the significant reduction in SMH of irradiated enamel observed after acid challenge during pH cycling in this study. Nevertheless, some authors have reported that irradiated enamel is not more susceptible to caries when adequate oral hygiene practices are employed, and that demineralization in irradiated enamel is quite similar to that in natural caries [14]. Additionally, another study reported no effect of irradiation on in vitro demineralization or in situ remineralization of human enamel [17].

This study aimed to investigate the effects of applying nano-HA, RI, and Cur on the SMH of irradiated enamel under acidic challenge. According to this study’s findings, these treatment agents influenced irradiated enamel microhardness; thus, the null hypothesis was rejected. Nano-HA treatment of irradiated enamel can effectively increase resistance to acid challenge, resulting in a final SMH level comparable to that of non-irradiated enamel. The effects of nano-HA on irradiated enamel have not been evaluated previously under pH-cycling conditions. Previously, several studies have demonstrated the high potential of nano-HA for remineralization of initial enamel lesions [25,26,29,47,51,52]. The high surface area of nanosized particles could impart greater mechanical strength to enamel [55]. Nano-HA may increase the mineral content of enamel, and its particles act as templates for the initiation of remineralization. This results in continuous attraction of a large amount of Calcium and Phosphate from the remineralization solution [26]. Due to the nano-size of its particles, nano-HA has a strong affinity to enamel and easily penetrates into the interprismatic enamel space [28,52]. In addition, the presence of acetone in a solution of nano-HA improves the dispersion of nano particles and their infiltrative ability [52]. As previous studies have demonstrated, the penetrated nano-HA particles could act as seeds for attracting and precipitating calcium and phosphate. There is a possibility that nano-HA can be incorporated into the porous tooth structure and may fill the micropores created in the irradiated enamel, thereby enhancing the remineralization of irradiated enamel under pH cycling.

The other protective treatment used in this study for irradiated enamel was the resin infiltration technique. This treatment was relatively effective in increasing resistance to acidic challenge. Although its final SMH was higher than that of untreated irradiated enamel, it was lower than that of the other treated groups and did not reach the level of non-irradiated enamel. RI arrests the progression of demineralization by capillary-driven penetration of a low-viscosity resin into the subsurface microporosities, followed by polymerization that mechanically seals diffusion pathways for acids and dissolved minerals [24] acting as a diffusion barrier to limit further demineralization [56]. The ability of RI to provide and maintain protection of demineralized enamel against an acidic challenge was demonstrated in previous studies [57]. In addition, RI was capable of occluding micro-porosities and microcracks in irradiated enamel [21]. It has been established that RI lacks remineralizing ability [24,58], and consequently, some researchers have attempted to use a combination of RI with a remineralizing agent. A previous study suggested that incorporating nano-HA into RI could be a promising method for resisting acid challenge to enamel [59]. Wu et al. applied calcium and fluoride compounds daily for 4 weeks after curing the infiltrated resin to enhance its remineralization activity. They investigated this combined intervention on the recovery of initial SMH of irradiated enamel and concluded that it had a higher, but no more substantial, effect than RI monotherapy [20]. However, their study did not evaluate their protective effect against acid challenge. It appears that the placement of remineralization compounds on the surface of the cured resin had no additional effect. Therefore, in the current study, the protective effect of the nano-HA and RI combination against acidic challenge during pH cycling was determined in a way that nano-HA was applied before the resin was used. This was assumed to result in compensation for the defective area mentioned above. When nano-HA penetrated the inner part of damaged enamel after HCl acid etching and was impregnated and sealed with RI, it appears to provide a more protective approach during acid challenge. In a previous research, applying nano-HA to acid-etched enamel could improve surface wettability and the penetration of a hydrophobic resin sealant [60]. Although the nano-HA and RI combination in this study exhibited a protective effect similar to that of nano-HA alone, its effect was considerably more substantial than that of RI monotherapy. These findings could reflect the strong remineralizing effect of nano-HA treatment, with or without RI, on irradiated enamel.

Following RT, the enamel surface becomes more uneven, with the appearance of small pits and microcracks [9,16,48]. This increased surface roughness and microporous structure could provide more niches for bacterial colonization and enhance bacterial adhesion [47,53]. Cur has a high potential to be used against pathogenic bacteria such as staphylococci, lactobacilli, and streptococci [32,61,62]. This could help prevent the progression of radiation caries in patients who have received RT. RI and nano-HA used in this study on irradiated enamel had no antibacterial effect. Hence, curcumin loaded on mesoporous silica nanoparticles was included in the experimental group in the current study, as it has unique properties that could benefit irradiated enamel. A previous study demonstrated a radioprotective effect of Cur applied during RT, preventing loss of mechanical strength and reducing wear resistance of irradiated enamel [18].

Radiation interacts with the inorganic and water content of enamel, inducing the formation of free radicals and hydrogen peroxide. This oxidation process could break the crystalline structure of teeth; the reduced enamel crystallinity results in decreased mechanical properties [18]. The use of curcumin, with its high antioxidant activity and its ability to scavenge reactive oxygen species [25,54], may prevent the destructive effects of free radicals on irradiated enamel, possibly enabling it to resist acidic attack during pH cycling. In this study, using Cur/MSN on irradiated enamel revealed a SMH_1_, but not statistically different, to that observed with nano-HA, with or without RI.

In the current study, MSNs were used to load curcumin, thereby enhancing its delivery into the micropores/cracks of irradiated enamel. MSN with a median particle diameter of 70 nm were synthesized. It has been reported that the infiltrated silica nanoparticles may provide a scaffold for the remineralization and mineral gain [29,63]. This mechanism may contribute to the beneficial effect of MSNs used in this study on enamel SMH after acid challenge. However, due to the larger size of MSN particles compared to nano-HA, their penetration into microcracks/pores of the enamel was less. These surface MSN particles were also visible in our SEM observation.

The strengths of this study include the use of a validated pH-cycling protocol to simulate post-radiation cariogenic challenges in RT patients. The study comprehensively assessed treatment effects using both surface microhardness and SEM micromorphology outcomes. In addition, multiple clinically relevant treatment strategies, nano-HA and RI as routine anticaries strategies, and Cur loaded on MSN as a novel experimental method, were directly compared under identical conditions, allowing evaluation of their relative effectiveness in restoring irradiated enamel resistance to acid challenge.

This study has some limitations. First, our RT protocol uses a dose of 60 Gy, while higher doses (70 Gy) may yield different effects on microhardness after pH cycling. Furthermore, despite the pathological similarities between naturally occurring enamel lesions and those induced by pH cycling [54], pH cycling cannot fully replicate the dynamic interplay among demineralization, remineralization, and microbial activity in the oral cavity [10,41]. It is also important to note that the anticaries treatments used in the current study altered the surface characteristics of irradiated enamel via different mechanisms. In addition to microhardness and micromorphology, the chemical composition and crystal structure of irradiated enamel are also influenced by various treatments, which were not analyzed in this study. In this study, the treatment agents were applied topically to the irradiated enamel for a brief period after RT was completed. In clinical practice, the treatment agents can be used before, during, or after RT sessions, or even for a longer period in subsequent sessions. Applying them earlier may provide better results and increase patient comfort. Therefore, further in vivo studies are required before definitive clinical recommendations can be made.

## Conclusion

Based on the microhardness and micromorphology findings, treatment with nano-HA and a combination of nano-HA with RI demonstrated significant potential to improve the resistance of the irradiated enamel surface to acidic challenges.

The study adequately addresses its teaching objectives by demonstrating that radiotherapy significantly compromises enamel surface microhardness and micromorphology, and that selected post-radiotherapy treatment approaches can wholly or partially restore enamel resistance to acidic challenge. The findings highlight nano-hydroxyapatite, with or without resin infiltration, as the most effective strategy for achieving microhardness values comparable to those of non-irradiated enamel.

## Supporting information

S1 File Plos one human participant research checklist. (DOCX)

S2 File Raw Data of the study. (XLSX)
